# First person – Sudipta Bar

**DOI:** 10.1242/dmm.037754

**Published:** 2018-11-21

## Abstract

First Person is a series of interviews with the first authors of a selection of papers published in Disease Models & Mechanisms (DMM), helping early-career researchers promote themselves alongside their papers. Sudipta Bar is first author on ‘Neuromuscular degeneration and locomotor deficit in a *Drosophila* model of mucopolysaccharidosis VII is attenuated by treatment with resveratrol’, published in DMM. Sudipta conducted the research described in this article while studying a PhD in Dr Rupak Datta's lab at the Indian Institute of Science Education and Research Kolkata, Nadia, India. He is now a Post Doc in the lab of Prof. Pankaj Kapahi at Buck Institute for Research on Aging, Novato, USA, investigating the molecular basis of neurodegeneration and how neurodegeneration affects memory.


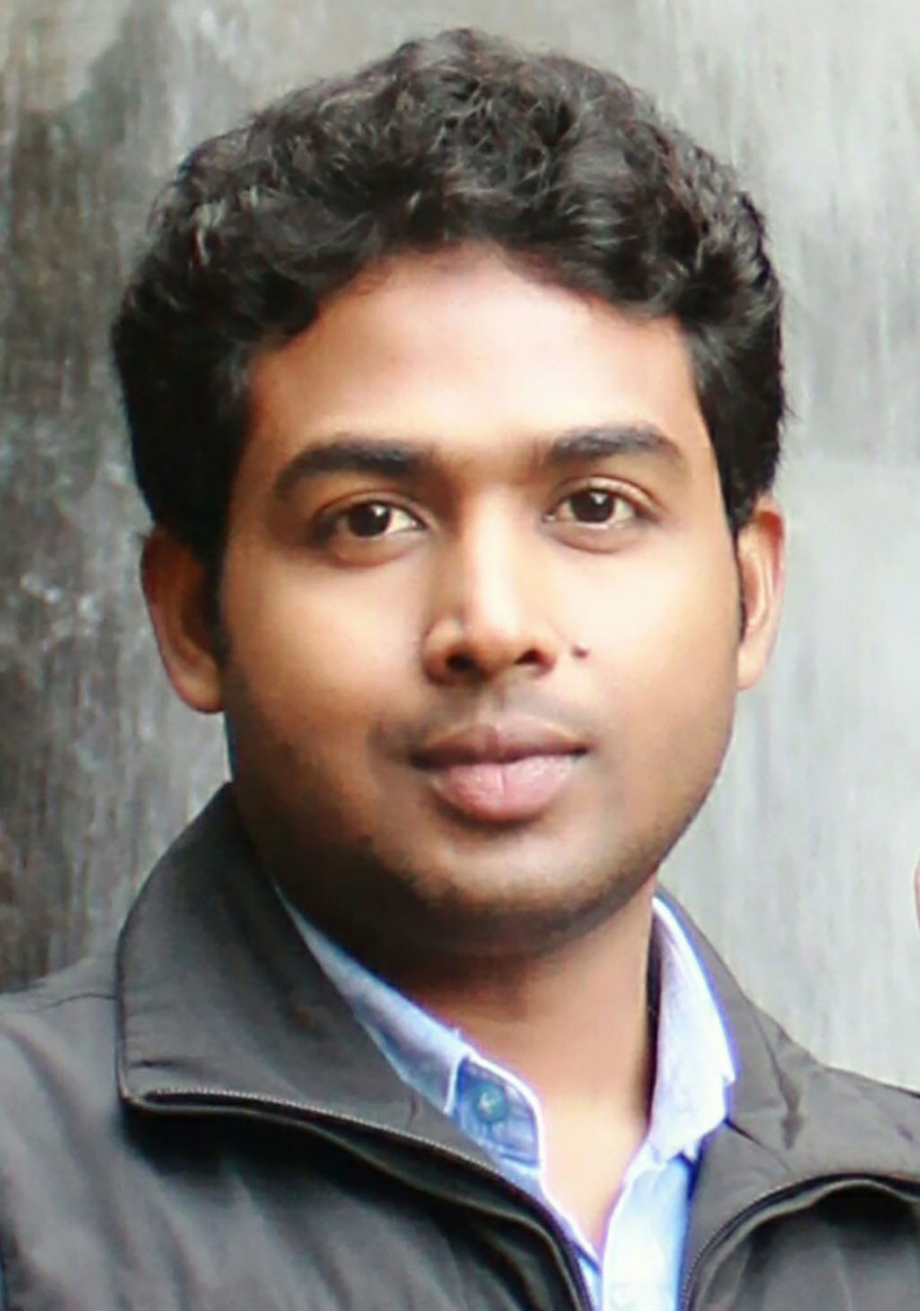


**Sudipta Bar**

**How would you explain the main findings of your paper to non-scientific family and friends?**

Mucopolysaccharidosis type VII (MPS VII) is a rare, hereditary lysosomal storage disease mostly accompanied with behavioral abnormality, organ failure and premature death. MPS VII is known to be caused by mutation in the β-glucuronidase gene. The only treatment is enzyme replacement therapy, which is an expensive, invasive and lifelong treatment procedure. Deep insight about the disease progression is also vaguely known and one of the limitations is the availability of a more suitable and handier disease model organism to study this human disease in a lab animal. We generated a fruit fly model of this disease by deleting the β-glucuronidase gene from the fruit fly genome. The fly model shows many similar phenotypes as a human patient. For example, premature death, short lifespan, storage of lysosome in brain and neurodegeneration. We used this model to study the disease in detail and found a major abnormality in dopaminergic neurons and muscle. Dopaminergic neuronal loss and muscle degeneration in this fly explained the basis of the disease-associated movement disability. We found that these defects could be corrected by treatment with resveratrol, thus providing a therapeutic lead. This novel MPS VII model holds the key to deeper exploration of the disease mechanism and drug discovery.

**What are the potential implications of these results for your field of research?**

The new fruit fly model, along with available genetic tools, will become more powerful to study the mechanistic details of MPS VII. The fly can be used in large-scale screening of drugs; for example, we observed the rescue of the disease phenotypes upon treatment with the drug resveratrol. This protective effect of resveratrol can be studied further as a potential MPS VII disease management strategy. In addition, the flies show abnormality in lysosome-mediated recycling processes in the brain along with neurodegeneration, which makes the model ideal to study the roles of lysosomes in neurodegeneration. Since the phenotypes among different types of MPS disorders are similar, the observations made in our MPS VII model may potentially be relevant in terms of a broader understanding and management of other closely related MPS disorders.

“This novel MPS VII model holds the key to deeper exploration of the disease mechanism and drug discovery.”

**What are the main advantages and drawbacks of the model system you have used as it relates to the disease you are investigating?**

The advantages of this fly model include that it is easily maintainable and in a short time period we can get thousands of flies to use in large-scale experiments, which is not possible with the existing mouse model of MPS VII. The fly genome can be easily manipulated with reporter constructs and genetic tools, which makes the study of the disease easier. This model is good for studying neurodegeneration, muscle degeneration and different visceral organ failures, but cannot study the bone-related abnormality found in MPS VII patients as the fly doesn't have skeletal system.

**Figure:**
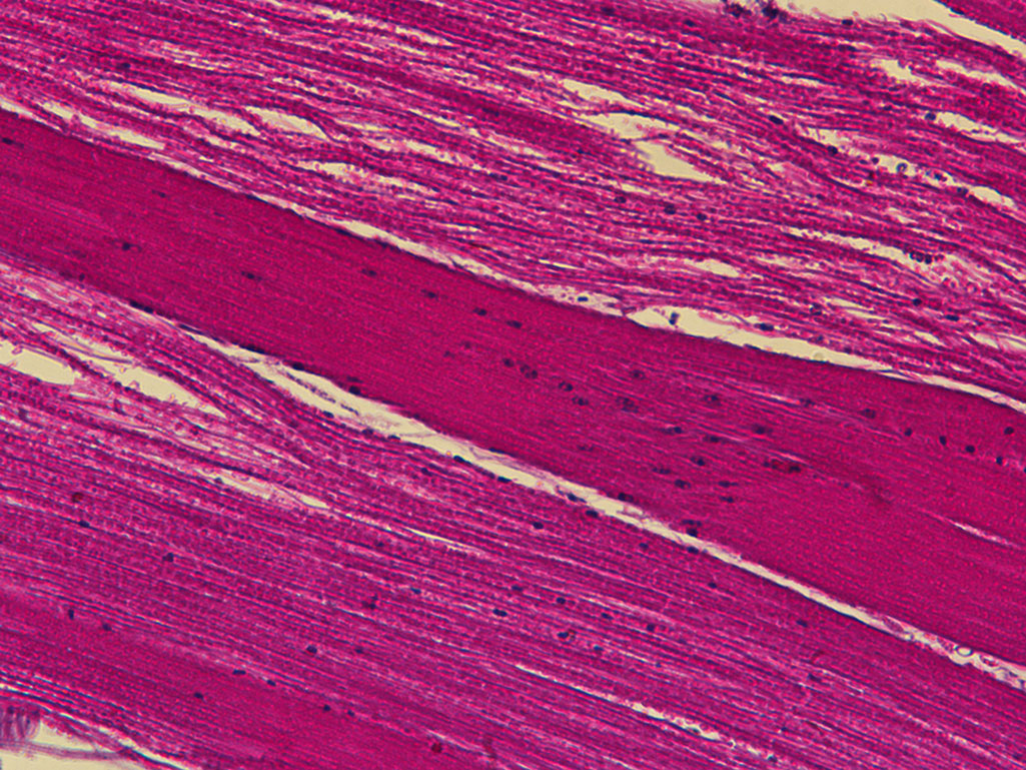
**Disrupted muscle fibres in *Drosophila* model of MPS VII**

**What has surprised you the most while conducting your research?**

Our model fly brain shows significant loss of dopaminergic neurons, which can be easily tracked by using whole-brain immunostaining. This phenotype was not known for this disease; this surprising result along with the other important novel finding in our study – muscle cell death – in the model fly can together explain the movement disability of MPS VII patients. We found these phenotypes to be rescued almost completely by resveratrol treatment. Almost complete rescue of these phenotypes with resveratrol was unexpected to us.

**Describe what you think is the most significant challenge impacting your research at this time and how will this be addressed over the next 10 years?**

The major challenge of finding a drug-based treatment procedure for this disease is insufficient knowledge about the disease. The genetic cause and abnormality in the corresponding metabolic pathway are well known for this disease but inadequate knowledge about disease progression and related molecular pathways need to be addressed in the next decade for the management of this rare disease. Our new model and already available genetic tools of *Drosophila* together will be useful in this study. 

**What changes do you think could improve the professional lives of early-career scientists?**

Mentors and institutions should encourage early-career scientists to carry forward their independent projects, to improve the power of thinking and hypothesizing a new idea. 

**What's next for you?**

After completing my PhD, I recently joined the Buck Institute for Research on Aging, CA, USA, as a postdoc, where I am studying neurodegeneration using the fruit fly as a model. In the future I have two interconnecting broad areas of interest; one is to study the mechanistic details of neurodegeneration in some disease conditions such as Alzheimer's disease, and the other is to explore the mechanism of memory formation in the brain.
